# Saffron Improves Epididymal Sperm Parameters in Rats Exposed to Cadmium

**DOI:** 10.5812/numonthly.12125

**Published:** 2013-11-04

**Authors:** Mohammad Hossein Asadi, Fariba Zafari, Arash Sarveazad, Mehdi Abbasi, Majid Safa, Morteza Koruji, Abazar Yari, Rafieh Alizadeh Miran

**Affiliations:** 1 Department of Anatomy, School of Medicine, Baqiyatallah University of Medical Sciences, Tehran, IR Iran; 2 Department of Anatomy, School of Medicine, Iran University of Medical Sciences, Tehran, IR Iran; 3 Department of Anatomy, School of Medicine, Tehran University of Medical Sciences, Tehran, IR Iran; 4 Cellular and Molecular Research Center, School of Allied Medical Sciences, Iran University of Medical Sciences, Tehran, IR Iran

**Keywords:** Crocus, Cadmium, Sperm Motility, Sperm Count, Rats

## Abstract

**Background:**

Cadmium (Cd) is known to cause various disorders in the testis. The general population may be exposed to Cd through ingestion of food and drinking water, inhalation of particulates from ambient air, tobacco smoke and ingestion of contaminated soil and dust. Saffron (Crocus sativus L.) is widely used as a food flavour, and has well known medicinal effects.

**Objectives:**

The aim of this study was to determine the effects of saffron on the results of semen parameters (sperm concentration, motility and viability in cauda of epididymis) in rats exposed to cadmium.

**Materials and Methods:**

Thirty Wistar male rats were divided into four groups. Cadmium chloride (1 mg/kg body weight) was injected intraperitoneally during 16 days at intervals of 48 hours between subsequent treatments. Crocus sativus L. (100 mg/kg b.w., IP) was pretreated in both control and cadmium-injected rats. Both control and cadmium-injected rats were pretreated with Crocus sativus L. (100 mg/kg b.w., IP). The animals were killed and their sperm count, motility, and vitality were evaluated.

**Results:**

Sperm parameters did not differ significantly between control and sham groups. Following contamination with cadmium, sperm count, motility and vitality were significantly decreased in comparison to control group (P < 0.05). In pretreated (saffron) group, the sperm parameters improved significantly in comparison with cadmium group (P ≤ 0.05). A significant decrease in sperm motility was observed in Cd-treated rats compared to the control rats. However, no significant changes were recorded by comparison of the control and saffron treated groups except for the sperm motility parameter.

**Conclusions:**

Saffron, as an antioxidant, is positively effective on sperm parameters in rats exposed with cadmium.

## 1. Background

Cadmium (Cd) is an extremely toxic, heavy metal used in industry. It is known to cause serious environmental and health effects including; damage to renal, hepatic, respiratory and reproductive systems, with the testes being particularly sensitive to its effects (1). The general population may be exposed to Cd through the ingestion of food and drinking water, inhalation of particulates, tobacco smoke, and the ingestion of contaminated soil and dust (2). Cd has been linked to osteomalacia, hepatotoxicity, renal toxicity, neurotoxicity as well as infertility and cancer (3, 4). Severe testicular hemorrhage, edema, and necrosis with destruction of seminiferous tubules, are the main testicular lesions which result from Cd exposure (5).

Cd produces oxidative stress by depleting glutathione and protein-bound sulfhydryl groups, which results in the enhanced production of reactive oxygen species such as superoxide ions, hydroxyl radicals, and hydrogen peroxide. These reactive oxygen species result in increased lipid peroxidation, excretion of urinary lipid metabolites, modulation of intracellular oxidized states, DNA damage, membrane damage, altered gene expression, and apoptosis (6). Some defence mechanisms can mitigate Cd-induced oxidative damage. Among these mechanisms, the antioxidants such as vitamins C, E, and L-Carnitine play a role as free radical scavengers (7, 8). It was reported that testis could be substantially protected from toxic effects of Cd by antioxidants treatment (7, 9).

Crocus sativus (saffron) is a perennial herb of the Iridaceae family with antioxidative prosperities (8, 10). Saffron has also been used for various applications such as sexual potential stimulant, antispasm, antidepression, sedative, and anti-inflammation (2, 11-13). In some countries such as India, Spain, and China, saffron has been used to treat infertility and impotence since long ago (14). Although a positive effect of some antioxidants against Cd- induced oxidative stress in testicular tissue has already been reported (3, 4, 12), the investigation of Crocus sativus L. effect on cadmium toxicity on sperm parameters has not been shown. Thus, the present study was performed to review the Crocus sativus L. antioxidant effects on sperm parameters of Cd-treated rats.

## 2. Objectives

In the light of information above, we undertook the present study to examine the beneficial effects of Crocus sativus L. on Cd-induced testicular damage. We evaluated the effect of Cd on semen parameters including sperm concentration, motility and viability in cauda of epididymis.

## 3. Materials and Methods

### 3.1. Chemicals

Cadmium chloride (Cdcl2) was obtained from Sigma (St. Louis, MO, USA). Crocus sativus L. stigmas were collected from Ghaen (Khorasan, Iran).

### 3.2. Animals

Thirty adult Wistar rats (4-4.5 months old), weighing 190-240 g were obtained from the Razi Research Center (Karaj, Iran).

### 3.3. Experimental Design

The animals were randomly divided into five groups, each consisting of six male mice including: 1) control group, 2) sham group: rats given normal saline (NS) (0.3 mL/kg body weight, intraperitoneal) for 16 days with an interval of 48 hours between subsequent treatments. 3) experimental 1 group (Crocus sativus L. group): rats given Crocus sativus L. (100 mg/kg b.w., IP) for 16 days with an interval of 48 hours between subsequent treatments, 4) experimental 2 group (Cd group): rats given Cd (1 mg/kg b.w., IP) for 16 days with an interval of 48 h between subsequent treatments, 5) experimental 3 group (Crocus sativus L. + Cd): rats given Crocus sativus L. + Cd in the same dose and time given to experimental groups of 1, and 2.

The animals were treated by Crocus sativus L. 1 hour prior to treatment with Cd. Cd administration dose was used according to a previous report (7). Rats were housed under 12-h light/ dark cycles. On the 17th day of the experiment, all animals were killed and samples were collected.

### 3.4. Preparation of Aqueous Saffron Extract

Saffron stigmas were collected from Ghaen (Khorasan, Iran). In the maceration method, 6 g of stigmas were macerated in 800 mL distilled water for three days. The mixture was subsequently filtered and concentrated under reduced pressure at 35ºC (14).

### 3.5. Sperm Collection

The cauda epididymis was excited, minced and incubated in a prewarmed petri dish containing 10 mL Hank’s balanced salt solution at 37°C. The spermatozoas were allowed to disperse into the buffer. After 20 min, the cauda of epididymis was removed, and the suspension was gently shaken to homogenize, and was analyzed under light microscope at a magnification of x400 (15, 16).

### 3.6. Sperm Parameters Assessment

#### 3.6.1. Sperm Count

For sperm counting, 500 μL of the sperm suspension was diluted with formaldehyde fixative (10% formalin in PBS). Approximately 10 μL from the diluted solution was transferred into a haemocytometer (Thoma, assistant Sondheim/Rhön, Germany) and let to stand for 7 min. Then the settled sperms were counted and evaluated per 250 small squares of a haemocytometer (17).

#### 3.6.2. Sperm Viability

Viability was assessed by eosin Y staining (5% in saline). Forty microlitersamples of the freshly sperm suspension were placed on a glass slide, mixed with 10 μL eosin and observed under a light microscope (x400 magnification). Live sperms remained unstained following staining; whereas, those that showed any pink or red coloration were classified as dead. At least 200 sperm were counted from each sample in ten fields of vision randomly, and the percentage of live sperms was recorded (18).

#### 3.6.3. Sperm Motility

To assess the percentage of motile sperm, the suspension was prepared by repipetting. A small aliquot (40 μL) of freshly liquefied semen was placed on a slide glass at 37˚C for film recording with a video microscope (Olympus, BX51, Germany). Randomly ten fields from each slide were recorded with camera for sperm motility assessment via analyzing the recorded films and counting progressive, nonprogressive and immotile spermatozoa graded from 1 to 2 (18, 19).

### 3.7. Statistical Analysis

All values are presented as mean ± SD. One-way analyses of variance and post-hoc Tukey test were performed to determine the differences among all groups for the whole parameters using the SPSS/PC computer program (version 17.0SS).

## 4. Results

### 4.1. Sperm Parameters Findings

**Figure 1.**
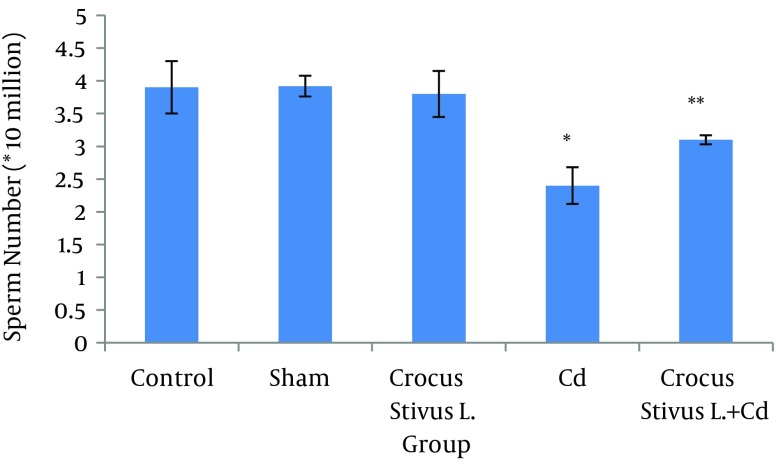
The Effect of Crocus Sativus L. on the Number of Cauda Epididymis Sperm in Cadmium-Treated Male Rats.

**Figure 2.**
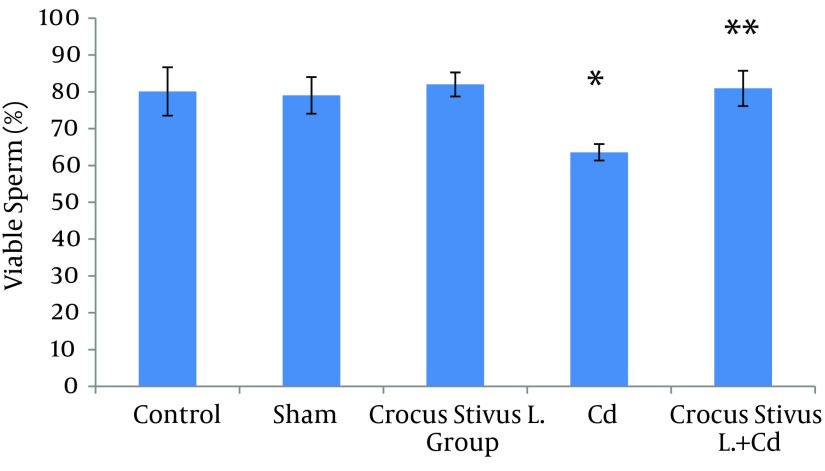
The Effect of Crocus Sativus L. on the Viability of Cauda Epididymis Sperm in Cadmium-Treated Male Rats.

**Figure 3.**
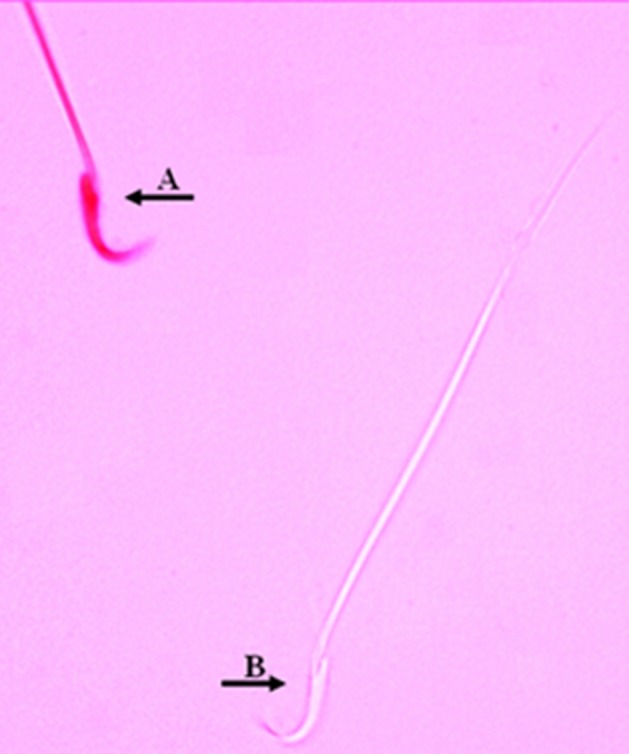
Sperm Viability: Dead (A) and Viable (B) Sperms.

**Figure 4.**
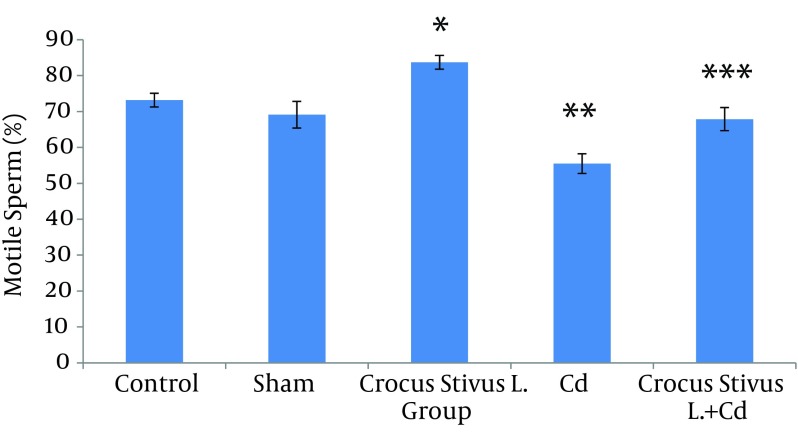
The Effect of Crocus Sativus L. on the Motility of Caudal Epididymis Sperm in Cadmium-Treated Male Rats.

**Figure 5.**
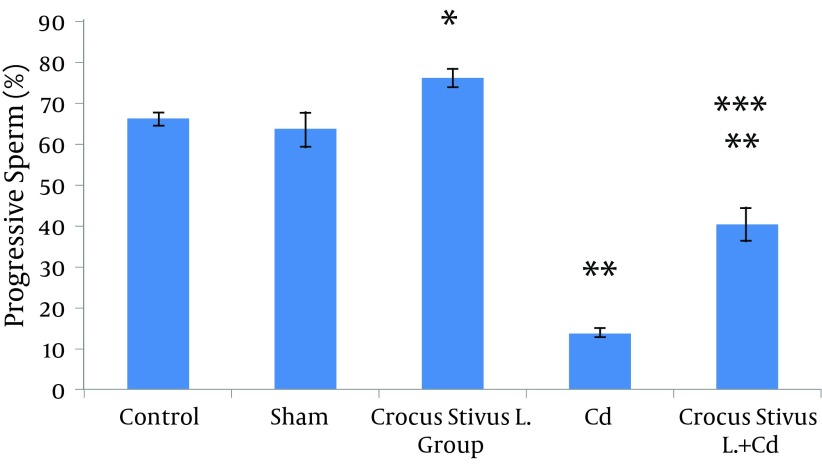
The Effect of Crocus Sativus L. on Progressive Motile of Cauda Epididymis Sperm in Cadmium-Treated Male Rats.

The differences in sperm parameters between control and sham groups were not significant. The sperm count, motility and vitality of rats in cadmium group decreased significantly in comparison to those of control and sham groups (P ≤ 0.05) (Figures 1-5 and Table 1). 

In pretreated group (group that received saffron), the sperm parameters improved significantly in comparison to those of the cadmium group (P ≤ 0.05). However, no significant changes were recorded by comparison of the control and treated groups except for the sperm motility parameter (Figures 1-5). A significant decrease in sperm motility was observed in Cd-treated rats, when compared to the control rats; however, there were no significant changes in other parameters between these two groups (P < 0.05; Figures 4 and 5, and Table 1). 

**Table 1.** The Effects of Saffron on the Sperm Motility in Adult Rat Exposed to Cadmium Groups (mean ± SD)

	Group 1 (Control)	Group 2 (Sham)	Group 3 (CSL)	Group 4 (Cadmium)	Group 5 (Cd + CSL.)
**Motility class a**	3.13 ± 0.27	5.63 ± 0.24	14.45 ± 0.78	0.00	1.00
**Motility class b**	62.83 ± 1.63	57.64 ± 4.48	61.39 ± 1.63	13.66 ± 0.87	39.03 ± 3.81
**Motility class c**	7.23 ± 0.78	5.84 ± 1.37	7.89 ± 0.88	41.84 ± 2.40	27.85 ± 1.67
**Motility class d**	26.80 ± 1.70	30.88 ± 3.72	16.28 ± 1.82	44.49 ± 2.68	32.10 ± 3.22
**Motile Sperm a + b + c**	73.18 ± 1.91	69.11 ± 3.72	83.73 ± 1.91	55.50 ± 2.73	67.88 ± 3.22
**Progressive Sperm a + b**	65.94 ± 1.66	63.28 ± 4.39	75.83 ± 2.36	13.96 ± 1.04	40.15 ± 3.81

## 5. Discussion

Cadmium is a toxic metal which promotes oxidative stress through the disruption of the antioxidative pro cess, and this contributes to the development of serious degenerative changes in various tissues, including the testes (20).

In our experiment, we have observed that Cd-treated rats exhibited a significant reduction in sperm count, motility and vitality. There were statistically significant differences between control and Cd-treated groups (Figures 1, 2, 4 and 5). Reduced number of sperm in a contaminated rat’s epididymis may be related to cell population decrease in the seminiferous tubules. Exposure to Cd can induce germ cell apoptosis, which may account for the current decline in male fertility (21, 22).

Increased apoptotic germ cells consisting of round spermatid and elongate spermatid were found in seminiferous tubules of Cd-treated rats. Moreover, higher Cd treatment resulted in severe necrosis of the seminiferous epithelium (8). Ramaiia and Pomerantseva reported that Cd causes cellular death in spermatocyte, and spermatogonium, which leads to male mice infertility (23). Another study conducted by Kasinathan et al. showed that Cd significantly decreased primary and secondary spermatocytes in the seminiferous tubules (24), and Foote reported that Cd reduced spermatogenesis in rabbits. In addition, Falsini et al. found that Cd reduced available spermatogenic cell population in treated animals (25).

Cadmium can directly inhibit primary Leydig cell testosterone levels, but the mechanism of this effect is not known (26). Our previous results showed that Cd leads to lower testosterone hormone production, which may be a secondary reason for reduction of sperm number in seminiferous tubules (unpublished paper).

Changes in sperm vitality and motility after Cd injection may be due to an increase in ROS levels in rat semen. Several lines of evidence indicate that ROS is involved in cadmium-induced testicular damage (26). In the past study we showed that Cd treatment induced an increase in lipid peroxidation of the testicular tissue as evidenced by an increase of TBARS levels. Various reports have shown that Cd induces oxidative stress by altering antioxidative status (9, 20, 27). Cd administration generates ROS in the cellular levels (28, 29) and associated to increase lipid peroxidation (30, 31). Hence Cd-induced ROS generation can increase lipid peroxidation which leads to testicular tissue damage. Indeed, a large proportion of infertile men have increased levels of seminal ROS (32, 33).

In this study we showed that after Crocus sativus L. administration, a significant increase in sperm motility was seen in CL group compared to control and sham groups (Figures 4 and 5). These changes may be due to antioxidant effects of saffron. Heidary et al. found that prescribing edible saffron is effective on increasing the average number and motility of sperms in nonsmoker infertile men with oligospermia (34). 

Our finding suggests that intraperitoneal administration of saffron in Cd-treated rats successfully increases the sperm quality. Although saffron has been shown to have antioxidant properties, these effects have not been investigated in Cd-pretreated rats. Our results showed that the sperm count, motility and viability in the presence of Crocus sativus L. were significantly improved compared to Cd only treated animals. Therefore positive changes in the sperm quality may be due to hydroxyl radical scavenging activity of saffron which inhibits lipid peroxidation. Our results suggest that saffron may increase spermatid cell viability through inhibition of oxidative stress and ROS production (35). By enhancing the antioxidant defence system of the cell, saffron reduces the oxidative stresses and increases the longevity of spermatozoids (36). These results regarding increasing the sperm count may be possibility caused by antiapoptotic effects of saffron. Furthermore, studies show that necrosis and apoptosis in the testes caused by Cd treatment decrease the level of antioxidizing enzymes (5).

It is possible that interference of saffron with free radical generation could be one of the causes of decline in Cd-induced damage. Therefore, saffron acts like an antioxidant in vivo, preventing the formation of free radicals and lipid peroxidation, hence preventing oxidant-induced apoptosis. Spermatogenic cells may be inhibited in the presence of Cd which can lead to spermatogenic cell population reduction in the seminiferous tubules (7). Similar studies have also reported treatment effects of antioxidants on testicular damages with Cd in animals (37, 38). A study by Kara et al. showed that a combination of antioxidants (melatonin, vitamin E and selenium) has streaking protective effect against cadmium-induced damage in testicular tissue (37).

Koyuturk et al. indicated that antioxidant treatment reduced oxidative stress, thus suggesting that antioxidant treatment may be a trigger in signalling a pathway for spermatogenic cells affected by Cd (3). On a survey performed on mice, saffron consumption with 100 mg/kg dosage during 20 days resulted in increased FSH, LH, and testosterone serum levels. Saffron may reduce hypophyseal–hypothalamus sensitivity to testosterone feedback control on LH secretion (39).

In the light of saffron antioxidants’ effects in biosynthesis of steroid hormones, it seems that saffron can affect the male sexual hormones concentration (40).

So, saffron administration improves sperm parameters in mice probably through increasing blood testosterone levels. Moreover, most changes that occur on testicle tissue and spermatogenesis process following saffron consumption are probably due to elevation of testosterone levels (39, 41). However, Safarinejad et al. reported that saffron administration for 26 weeks to the infertile men with idiopathic oligoasthenoteratozoospermia (OAT) had no effects on semen parameters (42).

Conclusions: The findings of this study showed that saffron may improve sperm count, motility and vitality in mice treated with cadmium. Therefore, saffron could be useful for the treatment of infertile men who were exposed to cadmium. The antioxidant effects of saffron may be a major reason for its positive impact on spermatic parameters. However, further studies are required to define its exact mechanism of action. Furthermore, the effect of saffron on cadmium-induced human infertility is needed to be more investigated.
